# Subphenotypes in Patients with Septic Shock Receiving Vitamin C, Hydrocortisone, and Thiamine: A Retrospective Cohort Analysis

**DOI:** 10.3390/nu11122976

**Published:** 2019-12-05

**Authors:** Won-Young Kim, Jae-Woo Jung, Jae Chol Choi, Jong Wook Shin, Jae Yeol Kim

**Affiliations:** Department of Internal Medicine, Chung-Ang University Hospital, Chung-Ang University College of Medicine, Seoul 06973, Korea; jwjung@cau.ac.kr (J.-W.J.); medics27@cau.ac.kr (J.C.C.); basthma@cau.ac.kr (J.W.S.); jykimmd@cau.ac.kr (J.Y.K.)

**Keywords:** ascorbic acid, hydrocortisone, leukocytes, septic shock, temperature, thiamine

## Abstract

This study aimed to identify septic phenotypes in patients receiving vitamin C, hydrocortisone, and thiamine using temperature and white blood cell count. Data were obtained from septic shock patients who were also treated using a vitamin C protocol in a medical intensive care unit. Patients were divided into groups according to the temperature measurements as well as white blood cell counts within 24 h before starting the vitamin C protocol. In the study, 127 patients included who met the inclusion criteria. In the cohort, four groups were identified: “Temperature ≥37.1 °C, white blood cell count ≥15.0 1000/mm^3^” (group A; *n* = 27), “≥37.1 °C, <15.0 1000/mm^3^” (group B; *n* = 30), “<37.1 °C, ≥15.0 1000/mm^3^” (group C; *n* = 35) and “<37.1 °C, <15.0 1000/mm^3^” (group D; *n* = 35). The intensive care unit mortality rates were 15% for group A, 33% for group B, 34% for group C, and 49% for group D (*p* = 0.051). The temporal improvement in organ dysfunction and vasopressor dose seemed more apparent in group A patients. Our results suggest that different subphenotypes exist among sepsis patients treated using a vitamin C protocol, and clinical outcomes might be better for patients with the hyperinflammatory subphenotype.

## 1. Introduction

Sepsis involves life-threatening organ dysfunction caused by a dysregulated host response to infection [1]. The global burden of sepsis is substantial, with an estimated 32 million cases and 5.3 million deaths per year [2]. In addition to the risk of short-term mortality, patients with sepsis experience various long-term complications and reduced quality of life. Thus, the cornerstones of sepsis treatment involve early identification, prompt antibiotic therapy, source control, and hemodynamic stability [3]. However, sepsis patients can still die of multiorgan dysfunction even if shock is prevented using these strategies.

Low-dose corticosteroids have been used as an adjuvant therapy for septic shock, as it downregulates the dysfunctional proinflammatory response and limits the anti-inflammatory response [4,5], increases adrenergic responsiveness [6], and preserves the endothelial glycocalyx [7]. Meanwhile, doses of corticosteroid in current guidelines do not consider the increased half-life of cortisol in the critically ill and may further increase central adrenocortical inhibition [8]. Two large randomized controlled trials have recently examined the effects of low-dose corticosteroids on mortality after septic shock [9,10], albeit with conflicting results. The APROCCHSS trial [10] reported that this treatment improved survival, whereas the ADRENAL trial [9] failed to detect a significant survival difference. The two trials had different inclusion–exclusion criteria, sources of sepsis, baseline therapies, and modes of hydrocortisone administration [11,12]. Another explanation may be that the current definition of sepsis captures a heterogeneous patient population. For example, Antcliffe et al. recently found a significant interaction between the previously identified sepsis response signatures (SRS) endotype and hydrocortisone therapy, demonstrating higher mortality in SRS2 patients treated using corticosteroids than in those treated using a placebo [13]. Nevertheless, most sepsis trials have focused on a one-size-fits-all approach, which may partially explain the inconsistent results from the aforementioned studies [9,10]. Therefore, novel methods of identifying subphenotypes among sepsis patients might help improve their management.

Vitamin C also limits the expression of proinflammatory cytokines, directly scavenges reactive oxygen species, and maintains endothelial barrier function [14,15]. Furthermore, there is evidence that vitamin C may act synergistically with corticosteroids [16,17] and that thiamine and vitamin C act to limit oxidative injury [18]. In septic shock patients, thiamine was associated with improved lactate clearance and a reduction in mortality [19]. These findings have led to recent observational studies, which demonstrated that sepsis patients experienced a substantial survival benefit after receiving vitamin C, hydrocortisone, and thiamine (which we will refer to as a “vitamin C protocol”) [20,21]. Several randomized controlled trials are currently underway to evaluate the effects of a vitamin C protocol on clinically important outcomes in sepsis. In the most recent CITRIS-ALI trial of patients with sepsis and acute respiratory distress syndrome (ARDS), a 96 h infusion of vitamin C compared with placebo did not significantly improve organ dysfunction scores or alter markers of inflammation and vascular injury [22]. However, the number of secondary outcomes including 28-day mortality, significantly favored vitamin C treatment. Moreover, differences in baseline characteristics of heterogeneous sepsis population may have influenced outcomes.

We hypothesize that subgroups exist within the septic phenotype receiving a vitamin C protocol, which will have variable physiological characteristics and clinical outcomes. In this study, body temperature and white blood cell count were used to classify these patients into novel subphenotypes.

## 2. Materials and Methods

### 2.1. Study Subjects, Study Design, and Treatment Protocol

This retrospective cohort study evaluated consecutive critically ill adults with sepsis or septic shock who were admitted to the medical intensive care unit (ICU) of an 835-bed university-affiliated tertiary hospital (Seoul, Korea) between September 2018 and August 2019. In September 2018, our institution adopted a vitamin C protocol as routine adjuvant therapy for septic shock due to experimental and emerging clinical data. The present study included consecutive patients who were treated with the vitamin C protocol, although patients were excluded if they were <19 years old, were not diagnosed with septic shock, and/or had a do-not-resuscitate order. Although this study did not evaluate the efficacy of the vitamin C protocol in septic shock, patients who were moribund and died within 24 h of receiving the protocol were also excluded.

Baseline demographics and physiological characteristics (i.e., vital signs, laboratory results) were compared between ICU survivors and non-survivors. In addition, the highest tympanic temperature measurements, as well as white blood cell counts from within 24 h before starting the vitamin C protocol, were compared between survivors and non-survivors. According to the temperature and white blood cell count, patients were divided into four groups. The primary study outcome was ICU mortality. The secondary outcomes included net fluid retention, vasopressor weaning, vasopressor-free days at day 28, ventilator weaning, ventilator-free days at day 28, hospital mortality, changes in the sequential organ failure assessment (SOFA) score [23] at day 4 relative to the start of the protocol, and changes in the norepinephrine equivalent dose and vasoactive-inotropic score at 24 h relative to the start of the protocol. Potential adverse effects of the vitamin C protocol were also analyzed. The study protocol was approved by the Institutional Review Board of Chung-Ang University Hospital (No. 1905-005-16264), and the requirement for written informed consent was waived due to the retrospective observational nature of the study.

The vitamin C protocol consisted of a combination of intravenous vitamin C (1.5 g every 6 h for 4 days), hydrocortisone (50 mg every 6 h for 7 days), and thiamine (200 mg every 12 h for 4 days) [20,21]. All patients were managed by adherence to therapeutic recommendations based on the surviving sepsis campaign guidelines and the lung-protective ventilation strategy [24,25].

### 2.2. Data Collection and Definitions

Baseline data were collected regarding age, sex, body mass index, comorbidities, cause of sepsis, presence of nosocomial infection, concurrent bacteremia, ARDS and/or septic cardiomyopathy, and the patients’ status within 24 h after ICU admission (mechanical ventilation, neuromuscular blockers, and/or renal replacement therapy). In addition, illness severity at the time of ICU admission was assessed by using the acute physiology and chronic health evaluation (APACHE) II score [26] and the SOFA score. Moreover, the time of septic shock onset, the time of starting the vitamin C protocol, and the vital signs and laboratory data from within 24 h before starting the vitamin C protocol were extracted. Intake and output of all fluids (urine volume, dialysis volume, drainage volume, and stool weight) were determined hourly for the first 4 days. The severity of organ dysfunction was assessed by calculating the SOFA score for the first 4 days. The hourly dosage of vasopressors was recorded as the norepinephrine equivalent dose [27] and the vasoactive-inotropic score [28]. We used the vasoactive-inotropic score to include vasopressin, which is commonly used in current practice. Sepsis and septic shock were defined using the third international consensus definitions for sepsis and septic shock (Sepsis-3) [1]. Twenty-two of 127 (17%) patients had a serum lactate level <2 mmol/L, although they were included in the study due to persisting hypotension requiring high-dose vasopressors. An immunocompromised status was diagnosed if there was an underlying disease or condition that affected the immune system (human immunodeficiency virus infection, malignancy, or severe neutropenia) or if immunosuppressive therapy was being administered. Nosocomial infections were defined as those occurring within 48 h of hospital admission. The consensus definition was used to identify ARDS [29]. Echocardiographic findings of septic cardiomyopathy were defined as left ventricular, right ventricular, or biventricular dysfunction [30]. The success of vasopressor weaning was defined as the ability of the patient to maintain normal pressure for 48 h without any vasopressor support. Ventilator weaning was identified based on the patient’s ability to breathe for 48 h without any form of ventilator support. Acute kidney injury was defined based on the KDIGO (Kidney Disease: Improving Global Outcomes) criteria [31]. Superinfection was diagnosed if a new microbiological infection occurred 48 h or more after admission.

### 2.3. Statistical Analysis

Continuous variables were presented as median (interquartile range [IQR]) or as mean ± standard deviation and were compared using the Mann-Whitney *U* test. Categorical variables were presented as number (percentage) and were compared using the chi-squared or Fisher’s exact test, as appropriate. The Kruskal-Wallis test was used to compare continuous variables among more than two groups. The cutoff temperature and white blood cell count values were the median values of study patients. Kaplan-Meier survival estimates were built stratified by initial temperature and white blood cell count to analyze their discriminating power in terms of predicting ICU mortality. All tests of significance were two-tailed, and differences were considered statistically significant at *p*-values of <0.05. All analyses were performed using IBM SPSS software (version 25.0; IBM Corp., Armonk, NY, USA).

## 3. Results

Among the 233 sepsis or septic shock patients admitted to ICU, 127 eligible patients were identified, including 84 patients (66%) who survived their ICU admission and 43 patients (34%) who died in the ICU. Seven patients with septic shock died within 24 h of receiving vitamin C protocol and were not included in the analysis (the baseline characteristics of these patients are detailed in Table S1).

### 3.1. Comparisons between Survivors and Non-Survivors

The patients’ characteristics from before starting the vitamin C protocol are shown in Table 1 according to survival status. No significant differences were observed between the survivors and non-survivors in terms of age, sex, body mass index, or comorbidities. Regarding the distribution of sepsis causes, the survivors were more likely to have urosepsis, while the non-survivors were more likely to have pneumonia. As expected, the non-survivors required more mechanical ventilation, neuromuscular blockers, and renal replacement therapy within 24 h of ICU admission and had significantly higher vasopressor doses. Before vitamin C protocol initiation, the survivors had a significantly higher median temperature (37.2 °C (IQR: 36.8–38.0 °C) vs. 36.9 °C (IQR: 36.4–37.8 °C); *p* = 0.01). The survivors tended to have non-significantly higher median values for white blood cell count (15.5 (IQR: 9.3–21.9) 1000/mm^3^ vs. 10.9 (IQR: 4.1–20.9) 1000/mm^3^; *p* = 0.08). Among other vital signs and laboratory data, the survivors had significantly higher PaO_2_/FiO_2_, while the non-survivors had significantly higher respiratory rate and serum lactate. Echocardiographic findings were available for 68 patients (54%), with no significant differences in left ventricular systolic function or the proportion of patients with septic cardiomyopathy. There was also no difference in median time from onset of shock to vitamin C protocol administration (5 (IQR: 1–12) h vs. 7 (IQR: 3–12) h; *p* = 0.27).

### 3.2. Baseline Characteristics and Clinical Outcomes between Study Groups

The median temperature and white blood cell count of study patients were 37.0 °C (IQR: 36.7–38.0 °C) and 14.4 (IQR: 8.0–21.8) 1000/mm^3^, respectively. Analysis of the baseline temperature and white blood cell count in the cohort found four study groups. Group A (*n* = 27; 21%) was characterized by a high presenting temperature (≥37.1 °C) with a high white blood cell count (≥15.0 1000/mm^3^). These patients could be referred to as the “hyperinflammatory” subphenotype. Similar to group A, group B (*n* = 30; 24%) also presented with a high temperature (≥37.1 °C) but with a low white blood cell count (<15.0 1000/mm^3^). Group C (*n* = 35; 28%) presented with a low temperature (<37.1 °C) but with a high white blood cell count (≥15.0 1000/mm^3^). Lastly, group D (*n* = 35; 28%) was characterized by low presenting temperature (<37.1 °C) with a low white blood cell count (<15.0 1000/mm^3^). These patients could be referred to as the “hypoinflammatory” subphenotype. When we included the patients who died within 24 h of receiving protocol, the median temperature and white blood cell count were 37.0 °C (IQR: 36.7–38.0 °C) and 14.9 (IQR: 8.1–21.9) 1000/mm^3^, respectively.

**Figure 1:**
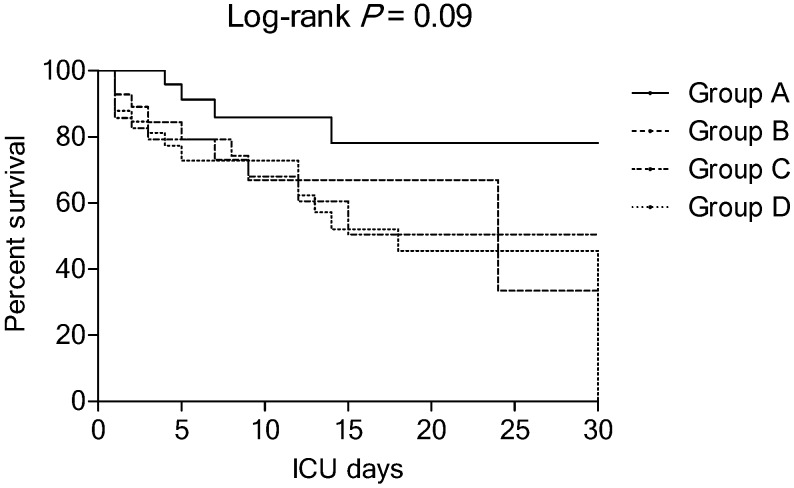
Kaplan-Meier curves for patients stratified according to the study group. ICU: Intensive care unit.

Table 2 shows the pre-vitamin C protocol characteristics of the patients according to study groups. In the cohort, group D patients had a significantly lower body mass index. There were no significant differences between the four groups in terms of the cause of sepsis, severity of illness (APACHE II and SOFA scores), patient’s status within 24 h after ICU admission, vital signs, and laboratory data except for temperature, white blood cell count, and PaO_2_/FiO_2_. In the cohort, group A and B patients had a significantly higher temperature than group C and D patients (*p* < 0.001). The group A and C patients had significantly higher white blood cell counts than group B and D patients (*p* < 0.001). The group B patients had significantly lower PaO_2_/FiO_2_ than the other groups. In group B patients, there was a significant delay in the median interval from shock onset to protocol administration (*p* = 0.03). Table 3 shows the clinical outcomes stratified according to study group. The group A patients (hyperinflammatory subphenotype) tended to have the lowest mortality rates and highest vasopressor and ventilator weaning rates. The Kaplan-Meier survival curves, stratified according to study group, are shown in Figure 1 (*p* = 0.09). When the 24 h non-survivors were included, the ICU mortality rates were 30% for group A, 43% for group B, 46% for group C, and 57% for group D (*p* = 0.18).

### 3.3. Physiological Characteristics between Study Groups

**Figure 2:**
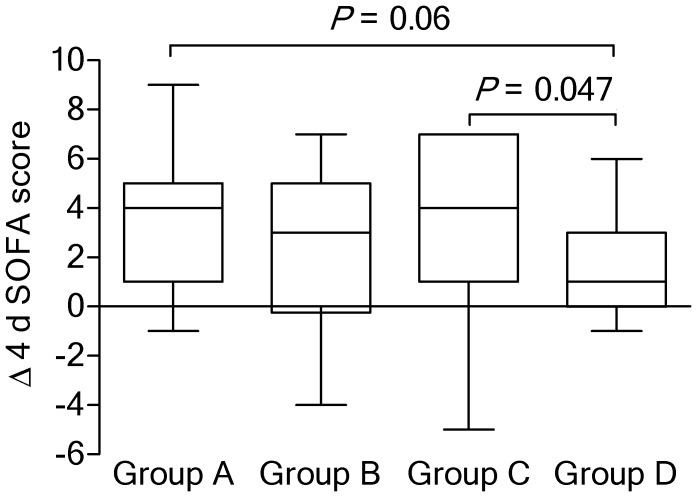
Median (interquartile range) changes in SOFA scores on day 4 relative to the scores on day 1, according to the study group. SOFA: Sequential Organ Failure Assessment.

There was no significant difference among the study groups in terms of median change in the SOFA score on day 4 relative to day 1 (*p* = 0.80; Kruskal-Wallis test). However, Δ 4 d SOFA scores tended to be higher in group A patients (4 (range 1–5)) compared with that of the group D patients (1 [range 0–3]; *p* = 0.06) (Figure 2). Interestingly and also unexpectedly, there was a statistically significant difference in improvement in SOFA score in group C patients, compared to group D patients (*p* = 0.047).

**Figure 3:**
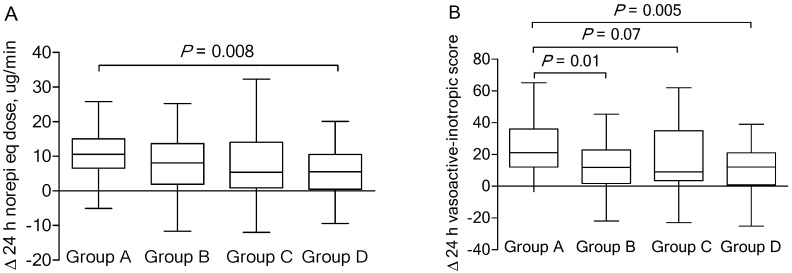
Median (interquartile range) change of the vasopressor dose (in norepinephrine equivalents (Norepi eq) or vasoactive-inotropic score) over the first 24 h according to the study group.

Figure 3 shows the median change of the vasopressor dose (in norepinephrine equivalents or vasoactive-inotropic score) over the first 24 h, according to the study group. Before the vitamin C protocol, no significant inter-group differences were observed in the median norepinephrine equivalent dose or the median vasoactive-inotropic score (Table 2). The norepinephrine equivalent doses decreased over time for all study groups, although there was no significant difference among the groups (*p* = 0.16; Kruskal-Wallis test). However, a significant inter-group difference was detected between group A and D patients (*p* = 0.008; Figure 3A). There was a significant difference among patients in terms of change in the vasoactive-inotropic score (*p* = 0.01; Kruskal-Wallis test), with significant inter-group differences between group A and B and group A and D patients (*p* = 0.01 and *p* = 0.005, respectively; Figure 3B). Other study group characteristics in the cohort are detailed in Table S2.

### 3.4. Septic Cardiomyopathy

Prior to vitamin C protocol administration, an echocardiography was performed in 68 of 127 (54%) patients. An echocardiographic finding of septic myocardial dysfunction was identified in 22 patients (Table S3). There was no significant difference in the proportion of patients with septic cardiomyopathy between the study groups (Table 2); however, the vasopressor weaning rate was highest (6/7; 86%), and the ICU mortality rate was lowest (1/7; 14%) in group A patients.

### 3.5. Adverse Events

Six of the 127 cohort patients (5%) developed acute kidney injury and needed renal replacement therapy throughout the study period. However, all of them were due to complications related to sepsis or underlying disease, and the relationship between toxicity and drug use was unclear. There was no significant difference between the study groups in terms of superinfection rates (15% vs. 20% vs. 9% vs. 17%; *p* = 0.62) (Table 3). Superinfection-related hospital mortality occurred in 5 (4%) patients (2 in group B and 3 in group D).

## 4. Discussion

The present study revealed subgroups in patients with septic shock who received the vitamin C protocol based on temperature and white blood cell count. We identified four subgroups of patients with considerable variations in their physiological differences. In addition, the groups exhibited different clinical outcomes to the vitamin C protocol, with the “hyperinflammatory” sepsis subphenotype potentially exhibiting a better clinical outcome. To the best of our knowledge, this is the first study to evaluate the subgroups in patients with septic shock receiving the vitamin C protocol.

Vitamin C exerts an anti-inflammatory effect by inhibiting the activation of nuclear factor kappa-B (NF-κB) [14,15], which modulates the transcription of several proinflammatory cytokines that promote antioxidant cellular injury [32] and endothelial dysfunction [33]. Vitamin C may also facilitate the production of catecholamines, vasopressin, and cortisol [34]. In this context, the primary anti-inflammatory action of corticosteroids involves suppressing the transcriptional activity of NF-κB and AP-1, which regulate the expression of cytokines, chemokines, inflammatory enzymes, cell adhesion molecules, coagulation factors, and receptors [35,36]. Furthermore, vitamin C may restore glucocorticoid receptor function [16], and corticosteroids increase cellular vitamin C uptake by increasing the expression of sodium-dependent vitamin C transporter-2 [17]. Moreover, thiamine exerts an anti-inflammatory effect by suppressing the oxidative stress-induced activation of NF-κB [37] and may act as a site-directed antioxidant [38]. Based on this information, our institution routinely started using the vitamin C protocol for septic shock in September 2018, as we believe that it is a combination of three safe, readily available, and inexpensive agents that target multiple pathways of the host’s response to infection. In this setting, septic cardiomyopathy is a reversible phenomenon that is caused by myocardial depressant factors and inefficient metabolism [39]. Hao et al. also demonstrated that vitamin C significantly decreased myocardial oxidant injury, attenuated apoptosis, and maintained the functional integrity of mitochondria by limiting calcium overload and inhibiting the opening of the mitochondrial permeability transition pore [40]. In the present study, patients with the highest vasopressor dose were weaned-off and survived the ICU stay with apparent echocardiographic improvements (Table S3). There is no biologically plausible explanation for this observation, although we observed marked improvements in the course of septic cardiomyopathy among patients with the hyperinflammatory subphenotype. Despite the relatively small sample sizes of our study, these findings are of great interest, and further studies are needed to evaluate the role of vitamin C in septic cardiomyopathy.

Several studies have revealed that hypothermia during infection is associated with an increased risk of mortality, whereas patients with fever are associated with a lower risk compared to those with normothermia [41], which also supports the presence of different phenotypes among patients with sepsis. Temperature may also reflect a patient’s underlying immunological state. Previous studies have investigated immunological differences between sepsis patients with and without fever based on their levels of pro- and anti-inflammatory cytokines [42,43], although the results failed to detect a significant difference in their cytokine profiles. There is a growing interest in immunomodulatory therapy, and accurate immunological phenotyping of sepsis patients is crucial [44]. Temperature itself may be useful for selecting immunosuppressive therapy in sepsis, even in the absence of specific cytokine abnormalities. In the present study, survivors had a significantly higher baseline temperature. However, there was heterogeneity in patients with an elevated baseline temperature (group A vs. group B). It is possible that the group A survivors tended to experience a gradual decrease in temperature (well-balanced inflammation); one can speculate, at least in part, why the group A patients exhibited better physiological characteristics and clinical outcomes than the group B patients. However, in the absence of a control group, it is not possible to draw associations between the vitamin C protocol’s immunomodulatory effect and the better clinical outcomes in group A patients. Further studies are required to test this hypothesis.

Bhavani et al. reported that “hyperthermic, slow resolvers” had the highest levels of inflammatory markers, such as erythrocyte sedimentation rate and C-reactive protein, as well as the highest incidence of leukocytosis [45]. These “hyperthermic, slow resolvers” may represent the hyperinflammatory subphenotype. Sweeny et al. also identified an “inflammopathic” subtype from sepsis datasets, which included high disease severity, high bandemia, and high mortality [46]. In the present study, we identified a hyperinflammatory subphenotype (group A patients) that was accompanied by elevated baseline values for temperature and white blood cell count. It is difficult to directly compare the hyperinflammatory phenotypes observed with those seen in the study by Sweeny et al., which used a clustering analysis to pool data from 14 transcriptomic datasets (*n* = 700). Meanwhile, group A patients appeared to be related to better clinical outcomes and survival and temporal improvements in the SOFA score and short-term vasopressor dose. We speculate that the hyperinflammatory subphenotype would respond better than the hypoinflammatory subphenotype to immunomodulatory therapies, such as corticosteroids or vitamin C protocol. However, the lack of a comparator group does not allow one to make any inferences about the relationship between the subgroups and the treatment response. Of note, neither the demographics, the cause of sepsis, or the severity indices distinguished the septic phenotypes from each other, since the specified variables had similar values across the phenotypes (Table 2). Our data suggest that phenotype is not just an indicator of severity of illness as measured by classical prognostic factors. There were statistically significant differences in SOFA score improvement in group C patients. There is no possible explanation for these findings, although they did not lead to better clinical outcomes.

Patients with multiorgan dysfunction who survive the first 2 weeks of sepsis often develop persistent inflammation/immunosuppression and catabolism syndrome (PICS) [47]. In this context, vitamin C has immune-enhancing properties [48,49] and also improves chemotaxis, enhances lymphocyte function, and assists in phagocytosis and intracellular killing of bacteria [50]. Moreover, low-dose short-term corticosteroid treatment was not associated with an increased risk of secondary infections in recent randomized controlled trials [9,10,51], which suggests that a vitamin C protocol may prevent PICS. However, the long-term effects of vitamin C, which is temporarily supplemented for 4 days, are only speculative since the human body does not store it [52]. In addition, 35 of the 43 non-survivors (81%) in our study died within 14 days after starting the vitamin C protocol, which was related to refractory shock and multiorgan dysfunction, and we could not evaluate the efficacy of the protocol in PICS.

The present study has several limitations. First, the single-center retrospective design is associated with various risks of bias. The small sample size and low power may be the cause of various non-significant results. Second, we do not have inflammatory markers such as cytokines to explain the immunological basis for these subphenotypes. Third, the vitamin C levels were not measured. There is a possibility that there were differences between the groups regarding vitamin C levels. Fourth, the present analysis was based on a series of arbitrary classifiers (temperature and white blood cell count). We chose the median values of study patients for sufficiently large groups for statistical analysis, but categorization based on either high or low levels, including normal ranges in either category, may contribute to some overlap and inadequate separation of the study patients. For example, the group with a temperature of ≥37.1°C may include patients with relatively normal temperatures. However, the median temperatures of group A and B patients were significantly higher than those of group C and D patients. Thus, this limitation does not undermine the original conclusion of the study. Fifth, the ICU mortality rate was relatively high when compared with that seen in previous studies [20,21,22,53]. We suggest that this observation results from a higher level of illness severity in study patients (all of them were on vasopressors and their median APACHE II scores reached almost 30). Moreover, the overall mortality rate of sepsis and septic shock in our institution is about 20%. Lastly, a control group would be needed to assess the efficacy of the vitamin C protocol in septic shock, although that was not one of the objectives of this hypothesis-generating study, which aimed to identify potential subgroups of patients with septic shock. This information might help improve our understanding regarding the heterogeneity of sepsis patients and may provide the basis for designing future randomized controlled trials.

## 5. Conclusions

In conclusion, we identified septic subphenotypes in patients receiving vitamin C protocol with varying pathophysiologies and clinical outcomes. These findings may help guide future interventional studies targeting the immune response in septic shock patients. However, independent validation in a larger sample is needed to address the present study’s findings.

## Figures and Tables

**Table 1:** Pre-vitamin C protocol characteristics according to the ICU survival status after septic shock.

Variable	Total (*n* = 127)	Survivors (*n* = 84)	Non-Survivors (*n* = 43)	*p*
Age, years	77 (68–83)	77 (68–82)	79 (68–84)	0.61
Male sex	75 (59)	47 (56)	28 (65)	0.32
Body mass index, kg/m^2^	21.3 (18.1–24.2)	21.3 (17.9–24.2)	21.1 (18.6–23.3)	0.98
Comorbidities				
Diabetes	44 (35)	28 (33)	16 (37)	0.66
Chronic heart failure	15 (12)	8 (10)	7 (16)	0.26
Chronic neurologic disease	38 (30)	26 (31)	12 (28)	0.72
Chronic lung disease	20 (16)	11 (13)	9 (21)	0.25
Liver cirrhosis	10 (8)	5 (6)	5 (12)	0.31
Chronic kidney disease	27 (21)	15 (18)	12 (28)	0.19
Malignancy	29 (23)	18 (21)	11 (26)	0.60
Immunocompromised	29 (23)	17 (20)	12 (28)	0.33
Nosocomial infection	49 (39)	29 (35)	20 (47)	0.19
Cause of sepsis				
Pneumonia	56 (44)	32 (38)	24 (56)	0.06
Urosepsis	35 (28)	30 (36)	5 (12)	0.004
Gastrointestinal/biliary	24 (19)	18 (21)	6 (14)	0.31
Skin/soft tissue	8 (6)	4 (5)	4 (9)	0.44
Concurrent bacteremia	36 (28)	25 (30)	11 (26)	0.62
ARDS at ICU admission	10 (8)	5 (6)	5 (12)	0.31
APACHE II score	28 (20–34)	25 (18–30)	31 (28–39)	<0.001
SOFA score	12 (10–14)	11 (10–12)	13 (12–15)	<0.001
Mechanical ventilation	87 (69)	48 (57)	39 (91)	<0.001
Neuromuscular blocker	35 (28)	15 (18)	20 (47)	0.001
Renal replacement therapy	41 (32)	11 (13)	30 (70)	<0.001
Vital signs and laboratory data				
Body temperature, °C	37.0 (36.7–38.0)	37.2 (36.8–38.0)	36.9 (36.4–37.8)	0.01
Mean arterial pressure, mmHg	60 (55–65)	60 (55–66)	58 (54–64)	0.20
Respiratory rate, breaths/min	28 (24–32)	27 (24–31)	30 (26–34)	0.03
PaO_2_/FiO_2_	214 (130–314)	240 (157–350)	147 (103–272)	0.007
Bicarbonate, mEq/L	19.2 (16.3–22.0)	19.4 (16.3–22.8)	19.0 (16.1–21.2)	0.59
Creatinine, mg/dL	1.4 (0.9–2.2)	1.3 (0.7–2.0)	1.7 (1.1–2.5)	0.11
White cell count, 1000/mm^3^	14.4 (8.0–21.8)	15.5 (9.3–21.9)	10.9 (4.1–20.9)	0.08
Total bilirubin, mg/dL	0.9 (0.5–1.6)	0.8 (0.5–1.6)	0.9 (0.5–1.9)	0.36
C-reactive protein, mg/L	135 (82–223)	137 (85–247)	133 (82–197)	0.74
Lactate, mmol/L	4.0 (2.5–7.0)	3.3 (2.3–6.4)	6.1 (3.9–8.5)	0.001
Norepi eq dose, ug/min	15.0 (9.4–21.3)	13.0 (5.6–18.7)	21.1 (12.9–32.4)	<0.001
Vasoactive-inotropic score	30.0 (18.6–48.7)	23.5 (14.1–44.1)	46.4 (26.7–74.7)	<0.001
Echocardiography (*n* = 47/21) ^1^				
Ejection fraction, %	56 (42–63)	57 (44–63)	55 (42–61)	0.55
Septic cardiomyopathy	22 (32)	13 (28)	9 (43)	0.22
Time from shock onset to vitamin C protocol, h	6 (2–12)	5 (1–12)	7 (3–12)	0.27

The data are presented as median (interquartile range) or number (percentage). ICU: Intensive care unit; ARDS: Acute respiratory distress syndrome; APACHE: Acute Physiology and Chronic Health Evaluation; SOFA: Sequential Organ Failure Assessment; PaO_2_: Arterial partial pressure of oxygen; FiO_2_: Fraction of inspired oxygen; Norepi eq: Norepinephrine equivalent. ^1^ No. of patients was 47 for survivors and 21 for non-survivors.

**Table 2:** Pre-vitamin C protocol patient characteristics according to the study group.

Variable	Group A (*n* = 27)	Group B (*n* = 30)	Group C (*n* = 35)	Group D (*n* = 35)	*p*
Age, years	77 (70–82)	77 (66–81)	78 (68–84)	79 (64–85)	0.88
Male sex	17 (63)	18 (60)	20 (57)	20 (57)	0.96
Body mass index, kg/m^2^	21.3 (17.9–23.3)	22.8 (19.6–25.2)	21.6 (19.6–25.1)	19.7 (17.5–22.3)	0.04
Comorbidities					
Diabetes	9 (33)	12 (40)	11 (31)	12 (34)	0.91
Chronic heart failure	2 (7)	4 (13)	5 (14)	4 (11)	0.87
Chronic neurologic disease	9 (33)	8 (27)	10 (29)	11 (31)	0.95
Chronic lung disease	7 (26)	2 (7)	8 (23)	3 (9)	0.09
Liver cirrhosis	1 (4)	1 (3)	5 (14)	3 (9)	0.38
Chronic kidney disease	3 (11)	6 (20)	8 (23)	10 (29)	0.41
Malignancy	6 (22)	6 (20)	8 (23)	9 (26)	0.96
Immunocompromised	5 (19)	6 (20)	7 (20)	11 (31)	0.56
Nosocomial infection	12 (44)	14 (47)	11 (31)	12 (34)	0.52
Cause of sepsis					
Pneumonia	11 (41)	15 (50)	13 (37)	17 (49)	0.68
Urosepsis	7 (26)	8 (27)	13 (37)	7 (20)	0.45
Gastrointestinal/biliary	7 (26)	6 (20)	5 (14)	6 (17)	0.69
Skin/soft tissue	0	4 (13)	1 (3)	3 (9)	0.17
Concurrent Bacteremia	5 (19)	8 (27)	14 (40)	9 (26)	0.29
ARDS at ICU admission	2 (7)	3 (10)	3 (9)	2 (6)	0.97
APACHE II score	30 (26–35)	28 (21–34)	28 (19–33)	26 (19–32)	0.45
SOFA score	11 (10–13)	13 (11–14)	12 (11–13)	12 (10–14)	0.15
Mechanical ventilation	21 (78)	23 (77)	22 (63)	21 (60)	0.30
Neuromuscular blocker	8 (30)	11 (37)	10 (29)	6 (17)	0.36
Renal replacement therapy	7 (26)	8 (27)	14 (40)	12 (34)	0.58
Vital signs and laboratory data					
Body temperature, °C	37.8 (37.4–38.2)	38.2 (37.8–38.5)	36.8 (36.4–36.9)	36.7 (36.5–37.0)	<0.001
Mean arterial pressure, mmHg	59 (57–66)	60 (52–64)	62 (56–68)	58 (53–64)	0.35
Respiratory rate, breaths/min	28 (26–33)	29 (27–34)	27 (24–32)	26 (24–31)	0.14
PaO_2_/FiO_2_	232 (152–314)	158 (99–208)	260 (162–340)	250 (120–351)	0.048
Bicarbonate, mEq/L	19.5 (17.1–22.4)	19.2 (16.3–20.7)	19.0 (14.4–20.9)	19.5 (17.4–22.8)	0.75
Creatinine, mg/dL	1.5 (0.7–1.9)	1.4 (1.0–1.9)	1.4 (1.0–2.6)	1.3 (0.7–2.2)	0.84
White cell count, 1000/mm^3^	21.9 (19.1–29.8)	8.1 (3.7–10.9)	21.9 (16.5–27.7)	8.1 (3.2–11.6)	<0.001
Total bilirubin, mg/dL	0.7 (0.4–1.3)	1.0 (0.6–1.9)	0.7 (0.5–2.6)	1.0 (0.6–1.5)	0.26
C-reactive protein, mg/L	135 (57–239)	150 (95–302)	140 (92–221)	115 (75–186)	0.20
Lactate, mmol/L	3.9 (2.6–7.0)	4.0 (3.1–6.1)	4.2 (2.3–6.9)	3.2 (2.1–7.2)	0.72
Norepi eq dose, ug/min	16.0 (10.2–19.1)	14.9 (10.4–21.1)	16.0 (9.6–28.3)	14.8 (6.5–20.7)	0.70
Vasoactive-inotropic score	32.0 (21.6–48.1)	25.9 (17.0–45.6)	38.0 (18.9–50.6)	27.0 (13.5–49.3)	0.71
Echocardiography (*n* = 17/15/21/15) ^1^					
Ejection fraction, %	56 (36–64)	54 (37–63)	56 (42–60)	59 (54–64)	0.40
Septic cardiomyopathy	7 (41)	6 (60)	6 (29)	3 (20)	0.52
Time from shock onset to vitamin C protocol, h	7 (3–12)	11 (4–20)	4 (1–8)	4 (1–8)	0.03

The data are presented as median (interquartile range) or number (percentage). ARDS: Acute respiratory distress syndrome; ICU: Intensive care unit; APACHE: Acute Physiology and Chronic Health Evaluation; SOFA: Sequential Organ Failure Assessment; PaO_2_: Arterial partial pressure of oxygen; FiO_2_: Fraction of inspired oxygen; Norepi eq: Norepinephrine equivalent. ^1^ No. of patients was 17 for Group A, 15 for Group B, 21 for Group C, and 15 for Group D.

**Table 3:** Clinical outcomes according to study group.

Variable	Group A (*n* = 27)	Group B (*n* = 30)	Group C (*n* = 35)	Group D (*n* = 35)	*p*
Net fluid retention ^1^, mL					
Day 1	1363 (183–2145)	2355 (1584–3169)	2215 (296–2954)	1839 (977–3058)	0.12
Day 2	674 (−13–1281)	723 (−252–1624)	650 (−12–1434)	1335 (476–2030)	0.34
Day 3	610 (−279–932)	230 (−388–750)	390 (−94–947)	560 (−138–1713)	0.43
Day 4	234 (−238–696)	280 (−255–850)	392 (−379–1178)	453 (−210–1139)	0.79
Vasopressor weaning	24 (89)	20 (69)	23 (66)	22 (63)	0.12
Vasopressor-free days at day 28	21.4 ± 9.0	17.2 ± 12.1	16.7 ± 12.3	15.3 ± 12.5	0.30
Ventilator weaning (*n* = 21/22/22/21) ^2,3^	15 (71)	14 (64)	8 (36)	6 (29)	0.01
Ventilator-free days at day 28	13.1 ± 11.1	13.4 ± 10.8	8.2 ± 11.2	5.6 ± 9.5	0.07
ICU mortality	4 (15)	10 (33)	12 (34)	17 (49)	0.051
Hospital mortality	6 (22)	13 (43)	15 (43)	19 (54)	0.09
Superinfection	4 (15)	6 (20)	3 (9)	6 (17)	0.62

The data are presented as median (interquartile range), mean ± standard deviation, or number (percentage). ICU: Intensive care unit. ^1^ The net fluid retention was determined as the difference between intake and output of all fluids (urine volume, dialysis volume, drainage volume, and stool weight). ^2^ Ventilator weaning was defined as the patient’s ability to breathe for 48 h without any ventilator support. ^3^ No. of patients was 21 for Group A, 22 for Group B, 22 for Group C, and 21 for Group D.

## Supplementary Materials

The following are available online at https://www.mdpi.com/2072-6643/11/12/2976/s1. Table S1: Pre-vitamin C protocol characteristics of the patients who died within 24 h of receiving protocol; Table S2: Change in physiological variables in the study groups; Table S3: Clinical characteristics including echocardiographic findings of 22 patients with septic cardiomyopathy.

## References

[1] Singer M., Deutschman C.S., Seymour C.W., Shankar-Hari M., Annane D., Bauer M., Bellomo R., Bernard G.R., Chiche J.D., Coopersmith C.M. (2016). The third international consensus definitions for sepsis and septic shock (sepsis-3). JAMA.

[2] Fleischmann C., Scherag A., Adhikari N.K., Hartog C.S., Tsaganos T., Schlattmann P., Angus D.C., Reinhart K. (2016). International Forum of Acute Care Trialists. Assessment of global incidence and mortality of hospital-treated sepsis. Current estimates and limitations. Am. J. Respir. Crit. Care Med..

[3] Castellanos-Ortega A., Suberviola B., Garcia-Astudillo L.A., Holanda M.S., Ortiz F., Llorca J., Delgado-Rodríguez M. (2010). Impact of the Surviving Sepsis Campaign protocols on hospital length of stay and mortality in septic shock patients: Results of a three-year follow-up quasi-experimental study. Crit. Care Med..

[4] Keh D., Boehnke T., Weber-Cartens S., Schulz C., Ahlers O., Bercker S., Volk H.D., Doecke W.D., Falke K.J., Gerlach H. (2003). Immunologic and hemodynamic effects of "low-dose" hydrocortisone in septic shock: A double-blind, randomized, placebo-controlled, crossover study. Am. J. Respir. Crit. Care Med..

[5] Marik P.E., Pastores S.M., Annane D., Meduri G.U., Sprung C.L., Arlt W., Keh D., Briegel J., Beishuizen A., Dimopoulou I. (2008). Recommendations for the diagnosis and management of corticosteroid insufficiency in critically ill adult patients: Consensus statements from an international task force by the American College of Critical Care Medicine. Crit. Care Med..

[6] Annane D., Bellissant E., Sebille V., Lesieur O., Mathieu B., Raphael J.C., Gajdos P. (1998). Impaired pressor sensitivity to noradrenaline in septic shock patients with and without impaired adrenal function reserve. Br. J. Clin. Pharmacol..

[7] Chappell D., Jacob M., Hofmann-Kiefer K., Bruegger D., Rehm M., Conzen P., Welsch U., Becker B.F. (2007). Hydrocortisone preserves the vascular barrier by protecting the endothelial glycocalyx. Anesthesiology.

[8] Téblick A., Peeters B., Langouche L., Van den Berghe G. (2019). Adrenal function and dysfunction in critically ill patients. Nat. Rev. Endocrinol..

[9] Venkatesh B., Finfer S., Cohen J., Rajbhandari D., Arabi Y., Bellomo R., Billot L., Correa M., Glass P., Harward M. (2018). Adjunctive glucocorticoid therapy in patients with septic shock. N. Engl. J. Med..

[10] Annane D., Renault A., Brun-Buisson C., Megarbane B., Quenot J.P., Siami S., Cariou A., Forceville X., Schwebel C., Martin C. (2018). Hydrocortisone plus fludrocortisone for adults with septic shock. N. Engl. J. Med..

[11] Venkatesh B., Cohen J. (2019). Why the adjunctive corticosteroid treatment in critically Ill patients with septic shock (ADRENAL) trial did not show a difference in mortality. Crit. Care Med..

[12] Annane D. (2019). Why my steroid trials in septic shock were “Positive”. Crit. Care Med..

[13] Antcliffe D.B., Burnham K.L., Al-Beidh F., Santhakumaran S., Brett S.J., Hinds C.J., Ashby D., Knight J.C., Gordon A.C. (2019). Transcriptomic signatures in sepsis and a differential response to steroids. from the VANISH randomized trial. Am. J. Respir. Crit. Care Med..

[14] Wilson J.X. (2009). Mechanism of action of vitamin C in sepsis: Ascorbate modulates redox signaling in endothelium. Biofactors.

[15] May J.M., Harrison F.E. (2013). Role of vitamin C in the function of the vascular endothelium. Antioxid. Redox Signal..

[16] Okamoto K., Tanaka H., Makino Y., Makino I. (1998). Restoration of the glucocorticoid receptor function by the phosphodiester compound of vitamins C and E, EPC-K1 (L-ascorbic acid 2-[3,4-dihydro-2,5,7,8-tetramethyl-2-(4,8,12-trimethyltridecyl)-2H-1-benzopyran-6 -yl hydrogen phosphate] potassium salt), via a redox-dependent mechanism. Biochem. Pharmacol..

[17] Fujita I., Hirano J., Itoh N., Nakanishi T., Tanaka K. (2001). Dexamethasone induces sodium-dependant vitamin C transporter in a mouse osteoblastic cell line MC3T3-E1. Br. J. Nutr..

[18] De Andrade J.A.A., Gayer C.R.M., Nogueira N.P.A., Paes M.C., Bastos V., Neto J., Alves S.C., Coelho R.M., da Cunha M.G.A.T., Gomes R.N. (2014). The effect of thiamine deficiency on inflammation, oxidative stress and cellular migration in an experimental model of sepsis. J. Inflamm..

[19] Woolum J.A., Abner E.L., Kelly A., Thompson Bastin M.L., Morris P.E., Flannery A.H. (2018). Effect of Thiamine administration on lactate clearance and mortality in patients with septic shock. Crit. Care Med..

[20] Marik P.E., Khangoora V., Rivera R., Hooper M.H., Catravas J. (2017). Hydrocortisone, vitamin c, and thiamine for the treatment of severe sepsis and septic shock: a retrospective before-after study. Chest.

[21] Kim W.Y., Jo E.J., Eom J.S., Mok J., Kim M.H., Kim K.U., Park H.K., Lee M.K., Lee K. (2018). Combined vitamin C, hydrocortisone, and thiamine therapy for patients with severe pneumonia who were admitted to the intensive care unit: Propensity score-based analysis of a before-after cohort study. J. Crit. Care.

[22] Fowler A.A., Truwit J.D., Hite R.D., Morris P.E., DeWilde C., Priday A., Fisher B., Thacker L.R., Natarajan R., Brophy D.F. (2019). Effect of vitamin C infusion on organ failure and biomarkers of inflammation and vascular injury in patients with sepsis and severe acute respiratory failure: the CITRIS-ALI randomized clinical trial. JAMA.

[23] Vincent J.L., Moreno R., Takala J., Willatts S., De Mendonca A., Bruining H., Reinhart C.K., Suter P.M., Thijs L.G. (1996). The SOFA (Sepsis-related Organ Failure Assessment) score to describe organ dysfunction/failure. On behalf of the working group on sepsis-related problems of the European society of intensive care medicine. Intensive Care Med..

[24] Rhodes A., Evans L.E., Alhazzani W., Levy M.M., Antonelli M., Ferrer R., Kumar A., Sevransky J.E., Sprung C.L., Nunnally M.E. (2017). Surviving sepsis campaign: international guidelines for management of sepsis and septic shock: 2016. Crit. Care Med..

[25] Brower R.G., Matthay M.A., Morris A., Schoenfeld D., Thompson B.T., Wheeler A., Acute Respiratory Distress Syndrome Network (2000). Ventilation with lower tidal volumes as compared with traditional tidal volumes for acute lung injury and the acute respiratory distress syndrome. N. Engl. J. Med..

[26] Knaus W.A., Draper E.A., Wagner D.P., Zimmerman J.E. (1985). APACHE II: A severity of disease classification system. Crit. Care Med..

[27] Patel B.M., Chittock D.R., Russell J.A., Walley K.R. (2002). Beneficial effects of short-term vasopressin infusion during severe septic shock. Anesthesiology.

[28] Gaies M.G., Gurney J.G., Yen A.H., Napoli M.L., Gajarski R.J., Ohye R.G., Charpie J.R., Hirsch J.C. (2010). Vasoactive-inotropic score as a predictor of morbidity and mortality in infants after cardiopulmonary bypass. Pediatr. Crit. Care Med..

[29] Ranieri V.M., Rubenfeld G.D., Thompson B.T., Ferguson N.D., Caldwell E., Fan E., Camporota L., Slutsky A.S., ARDS Definition Task Force (2012). Acute respiratory distress syndrome: The Berlin definition. JAMA.

[30] Griffee M.J., Merkel M.J., Wei K.S. (2010). The role of echocardiography in hemodynamic assessment of septic shock. Crit. Care Clin..

[31] Kellum J.A., Lameire N., KDIGO AKI Guideline Work Group (2013). Diagnosis, evaluation, and management of acute kidney injury: A KDIGO summary (Part 1). Crit. Care.

[32] Oudemans-van Straaten H.M., Spoelstra-de Man A.M., de Waard M.C. (2014). Vitamin C revisited. Crit. Care.

[33] Dhar-Mascareno M., Carcamo J.M., Golde D.W. (2005). Hypoxia-reoxygenation-induced mitochondrial damage and apoptosis in human endothelial cells are inhibited by vitamin C. Free Radic. Biol. Med..

[34] Carr A.C., Shaw G.M., Fowler A.A., Natarajan R. (2015). Ascorbate-dependent vasopressor synthesis: A rationale for vitamin C administration in severe sepsis and septic shock?. Crit. Care.

[35] Busillo J.M., Cidlowski J.A. (2013). The five Rs of glucocorticoid action during inflammation: Ready, reinforce, repress, resolve, and restore. Trends Endocrinol. Metab..

[36] Cain D.W., Cidlowski J.A. (2017). Immune regulation by glucocorticoids. Nat. Rev. Immunol..

[37] Manzetti S., Zhang J., van der Spoel D. (2014). Thiamin function, metabolism, uptake, and transport. Biochemistry.

[38] Gibson G.E., Zhang H. (2002). Interactions of oxidative stress with thiamine homeostasis promote neurodegeneration. Neurochem. Int..

[39] Krishnagopalan S., Kumar A., Parrillo J.E., Kumar A. (2002). Myocardial dysfunction in the patient with sepsis. Curr. Opin. Crit. Care.

[40] Hao J., Li W.W., Du H., Zhao Z.F., Liu F., Lu J.C., Yang X.C., Cui W. (2016). Role of vitamin C in cardioprotection of ischemia/reperfusion injury by activation of mitochondrial KATP channel. Chem. Pharm. Bull..

[41] Rumbus Z., Matics R., Hegyi P., Zsiboras C., Szabo I., Illes A., Petervari E., Balasko M., Marta K., Miko A. (2017). Fever Is Associated with Reduced, Hypothermia with increased mortality in septic patients: A meta-analysis of clinical trials. PLoS ONE.

[42] Marik P.E., Zaloga G.P. (2000). Hypothermia and cytokines in septic shock. Norasept II Study Investigators. North American study of the safety and efficacy of murine monoclonal antibody to tumor necrosis factor for the treatment of septic shock. Intensive Care Med..

[43] Wiewel M.A., Harmon M.B., van Vught L.A., Scicluna B.P., Hoogendijk A.J., Horn J., Zwinderman A.H., Cremer O.L., Bonten M.J., Schultz M.J. (2016). Risk factors, host response and outcome of hypothermic sepsis. Crit. Care.

[44] Hotchkiss R.S., Monneret G., Payen D. (2013). Immunosuppression in sepsis: A novel understanding of the disorder and a new therapeutic approach. Lancet Infect. Dis..

[45] Bhavani S.V., Carey K.A., Gilbert E.R., Afshar M., Verhoef P.A., Churpek M.M. (2019). Identifying novel sepsis subphenotypes using temperature trajectories. Am. J. Respir. Crit. Care Med..

[46] Sweeney T.E., Azad T.D., Donato M., Haynes W.A., Perumal T.M., Henao R., Bermejo-Martin J.F., Almansa R., Tamayo E., Howrylak J.A. (2018). Unsupervised analysis of transcriptomics in bacterial sepsis across multiple datasets reveals three robust clusters. Crit. Care Med..

[47] Mira J.C., Gentile L.F., Mathias B.J., Efron P.A., Brakenridge S.C., Mohr A.M., Moore F.A., Moldawer L.L. (2017). Sepsis pathophysiology, chronic critical illness, and Persistent Inflammation-Immunosuppression and Catabolism Syndrome. Crit. Care Med..

[48] Huijskens M.J., Walczak M., Sarkar S., Atrafi F., Senden-Gijsbers B.L., Tilanus M.G., Bos G.M., Wieten L., Germeraad W.T. (2015). Ascorbic acid promotes proliferation of natural killer cell populations in culture systems applicable for natural killer cell therapy. Cytotherapy.

[49] Manning J., Mitchell B., Appadurai D.A., Shakya A., Pierce L.J., Wang H., Nganga V., Swanson P.C., May J.M., Tantin D. (2013). Vitamin C promotes maturation of T-cells. Antioxid. Redox Signal..

[50] Wilson J.X. (2013). Evaluation of vitamin C for adjuvant sepsis therapy. Antioxid. Redox Signal..

[51] Keh D., Trips E., Marx G., Wirtz S.P., Abduljawwad E., Bercker S., Bogatsch H., Briegel J., Engel C., Gerlach H. (2016). Effect of hydrocortisone on development of shock among patients with severe sepsis: The hypress randomized clinical trial. JAMA.

[52] Naidu K.A. (2003). Vitamin C in human health and disease is still a mystery? An overview. Nutr. J..

[53] Shin T.G., Kim Y.J., Ryoo S.M., Hwang S.Y., Jo I.J., Chung S.P., Choi S.H., Suh G.J., Kim W.Y. (2019). Early vitamin C and thiamine administration to patients with septic shock in emergency departments: Propensity score-based analysis of a before-and-after cohort study. J. Clin. Med..

