# Supplementary material for: Subphenotypes in Patients with Septic Shock Receiving Vitamin C, Hydrocortisone, and Thiamine: A Retrospective Cohort Analysis
Source: Nutrients. 2019 Dec 5;11(12):2976. doi: 10.3390/nu11122976 (PMC6950320; doi:10.3390/nu11122976)
Supplement: Supplementary file 1 [file nutrients-11-02976-s001.pdf]

Table S1. Pre-vitamin C protocol characteristics of the patients who died within 24 h of receiving protocol.

Variable	Total (n = 7)
Age, years	83 (70–86)
Male sex	3 (43)
Body mass index, kg/m^2	21.3 (19.0–24.8)
Comorbidities	
Diabetes	3 (43)
Chronic heart failure	0
Chronic neurologic disease	0
Chronic lung disease	2 (29)
Liver cirrhosis	0
Chronic kidney disease	4 (57)
Malignancy	3 (43)
Immunocompromised	3 (43)
Nosocomial infection	5 (71)
Cause of sepsis	
Pneumonia	2 (29)
Urosepsis	2 (29)
Gastrointestinal/biliary	4 (57)
Skin/soft tissue	0
Concurrent bacteremia	2 (29)
ARDS at ICU admission	0
APACHE II score	39 (37–43)
SOFA score	14 (13–16)
Mechanical ventilation	7 (100)
Neuromuscular blocker	0
Renal replacement therapy	6 (86)
Vital signs & laboratory data	
Body temperature, °C	36.9 (36.3–37.9)
Mean arterial pressure, mmHg	40 (30–43)
Respiratory rate, breaths/min	30 (28–32)
PaO_2/FiO_2	238 (181–339)
Bicarbonate, mEq/L	12.2 (11.8–13.5)
Creatinine, mg/dL	3.5 (1.8–3.8)
White cell count, 1000/mm^3	20.0 (13.9–25.6)
Total bilirubin, mg/dL	0.8 (0.6–0.8)
C-reactive protein, mg/L	105 (74–133)
Lactate, mmol/L	17.0 (8.4–20.0)
Norepi eq dose, ug/min	42.9 (32.6–62.7)
Vasoactive-inotropic score	81.4 (63.6–138.0)
Day 1 net fluid retention [1], mL	2969 (2752–4005)
Time from shock onset to vitamin C protocol, h	7 (4–12)

The data are presented as median (interquartile range) or number (percentage). ARDS: acute respiratory distress syndrome; ICU: intensive care unit; APACHE: Acute Physiology and Chronic Health Evaluation; SOFA: Sequential Organ Failure Assessment; PaO_2: arterial partial pressure of oxygen; FiO_2: fraction of inspired oxygen; Norepi eq: norepinephrine equivalent. [1] The net fluid retention was determined as the difference between intake and output of all fluids (urine volume, dialysis volume, drainage volume, and stool weight).

Table S2. Change in physiological variables in the study groups.

Variable	Group A (n = 27)	Group B (n = 30)	Group C (n = 35)	Group D (n = 35)	*P*
**Absolute value**					
Norepi eq dose, ug/min					
Baseline	16.0 (10.2–19.1)	14.9 (10.4–21.1)	16.0 (9.6–28.3)	14.8 (6.5–20.7)	0.70
3 h	13.1 (6.9–19.4)	14.4 (10.5–24.8)	16.1 (9.5–22.4)	10.7 (7.3–22.1)	0.72
6 h	10.8 (4.4–19.1)	14.2 (9.5–24.6)	14.0 (6.9–27.9)	10.8 (5.2–21.6)	0.46
12 h	7.2 (2.9–13.0)	10.5 (6.6–18.0)	10.5 (4.1–20.5)	7.9 (2.1–22.1)	0.37
24 h	5.4 (0–9.1)	6.1 (2.1–12.0)	4.1 (0.5–16.9)	4.0 (0–20.1)	0.61
Vasoactive-inotropic score					
Baseline	32.0 (21.6–48.1)	25.9 (17.0–45.6)	38.0 (18.9–50.6)	27.0 (13.5–49.3)	0.71
3 h	28.0 (16.1–43.6)	27.0 (19.0–48.1)	35.0 (18.4–53.8)	31.5 (13.0–52.4)	0.93
6 h	19.7 (11.0–39.9)	29.2 (17.0–46.0)	33.7 (13.4–57.2)	25.3 (10.0–62.7)	0.62
12 h	16.1 (5.9–32.4)	22.9 (15.3–38.1)	25.0 (12.0–51.2)	20.0 (5.0–57.0)	0.46
24 h	8.0 (0–20.0)	11.7 (5.8–24.0)	8.3 (3.3–41.3)	9.7 (2.0–50.6)	0.42
Day 2 (n = 27/30/35/34) [1]					
Vasopressor use	25 (93)	28 (93)	34 (97)	32 (94)	0.88
Mean arterial pressure, mmHg	62 (57–67)	64 (60–68)	62 (55–68)	64 (52–68)	0.90
PaO_2/FiO_2	248 (167–314)	217 (149–254)	219 (162–333)	227 (150–369)	0.79
Creatinine, mg/dL	1.1 (0.7–1.8)	1.3 (1.0–2.2)	1.3 (0.7–1.8)	1.2 (0.6–2.0)	0.54
White cell count, 1000/mm^3	20.7 (16.2–32.3)	12.0 (8.0–15.8)	20.7 (15.9–26.7)	8.3 (4.4–13.5)	<0.001
Total bilirubin, mg/dL	0.7 (0.4–1.6)	1.0 (0.5–2.4)	0.8 (0.5–1.9)	0.8 (0.5–2.3)	0.41
C-reactive protein, mg/L	233 (130–279)	254 (194–317)	163 (120–287)	146 (100–247)	0.009
Lactate, mmol/L	2.7 (2.0–4.4)	4.4 (2.1–9.2)	2.7 (1.8–6.8)	3.9 (2.2–6.0)	0.40
SOFA score	10 (9–13)	12 (9–14)	11 (10–14)	12 (9–16)	0.33
Day 3 (n = 27/25/31/29) [2]					
Vasopressor use	16 (59)	16 (64)	25 (81)	22 (76)	0.25
Mean arterial pressure, mmHg	62 (55–74)	66 (57–72)	64 (60–72)	60 (53–72)	0.78
PaO_2/FiO_2	286 (223–354)	236 (194–282)	197 (170–321)	297 (184–382)	0.27
Creatinine, mg/dL	1.0 (0.6–1.8)	1.2 (0.8–1.6)	1.2 (0.6–1.4)	0.9 (0.5–2.2)	0.84
White cell count, 1000/mm^3	18.7 (12.5–36.4)	12.6 (8.9–18.3)	18.5 (13.9–24.0)	9.7 (6.5–12.9)	<0.001

Total bilirubin, mg/dL	0.6 (0.4–1.5)	0.7 (0.5–2.4)	0.8 (0.5–1.7)	1.0 (0.5–2.4)	0.35
C-reactive protein, mg/L	137 (74–258)	215 (150–282)	174 (87–232)	135 (68–233)	0.11
Lactate, mmol/L	2.3 (1.8–3.0)	2.2 (1.7–4.2)	2.2 (1.8–4.3)	3.0 (2.3–3.5)	0.44
SOFA score	10 (5–12)	9 (7–12)	11 (8–13)	11 (8–15)	0.16
Day 4 (n = 27/23/25/25) [3]					
Vasopressor use	11 (41)	11 (48)	13 (52)	15 (60)	0.57
Mean arterial pressure, mmHg	72 (59–77)	68 (62–73)	66 (60–74)	63 (54–73)	0.50
PaO_2/FiO_2	270 (171–319)	231 (183–263)	225 (167–311)	311 (198–364)	0.12
Creatinine, mg/dL	0.8 (0.5–1.6)	1.1 (0.7–1.2)	0.9 (0.5–1.2)	0.7 (0.6–1.8)	0.83
White cell count, $1000/mm^3$	15.8 (13.1–27.8)	12.0 (8.4–15.0)	16.1 (12.3–18.1)	8.4 (6.5–13.2)	<0.001
Total bilirubin, mg/dL	0.6 (0.4–1.4)	0.9 (0.4–1.9)	0.8 (0.5–1.6)	0.8 (0.5–3.0)	0.55
C-reactive protein, mg/L	63 (38–155)	127 (85–174)	85 (42–154)	76 (42–188)	0.27
Lactate, mmol/L	1.9 (1.5–2.9)	2.8 (1.6–4.1)	2.0 (1.7–2.4)	2.6 (1.8–3.7)	0.29
SOFA score	8 (6–11)	10 (6–13)	10 (5–12)	10 (7–14)	0.32
**Change relative to baseline values**					
SOFA score, day 4	4 (1–5)	3 (0–5)	4 (1–7)	1 (0–3)	0.80
Lactate, day 4	1.9 (0.2–5.4)	0.7 (-0.8–2.0)	1.7 (0.4–3.9)	1.2 (-0.1–4.0)	0.051
Norepi eq dose, 24 h	10.6 (7.0–15.0)	8.1 (2.1–13.6)	5.4 (1.4–14.0)	5.5 (1.3–10.5)	0.16
Vasoactive-inotropic score, 24 h	21.1 (12.0–33.0)	11.9 (2.7–22.3)	9.0 (3.9–34.0)	12.0 (0.9–21.0)	0.01

The data are presented as median (interquartile range) or number (percentage). Norepi eq: norepinephrine equivalent; PaO_2: arterial partial pressure of oxygen; FiO_2: fraction of inspired oxygen; SOFA: Sequential Organ Failure Assessment. [1] No. of patients was 27 for Group A, 30 for Group B, 35 for Group C, and 34 for Group D. [2] No. of patients was 27 for Group A, 25 for Group B, 31 for Group C, and 29 for Group D. [3] No. of patients was 27 for Group A, 23 for Group B, 25 for Group C, and 25 for Group D.

Table S3. Clinical characteristics including echocardiographic findings of 22 patients with septic cardiomyopathy.

Group	Age / sex	Cause of sepsis	Norepi eq dose, μg/min	VIS	Echocardiographic findings	Echo improved	Vasopressor weaning	ICU mortality
A	69 / M	Pneumonia	14.0	47	Severe LV systolic dysfunction	N/A	Yes	No
A	95 / M	Urosepsis	77.1	121	Moderate LV systolic dysfunction, RV systolic dysfunction	Yes	Yes	No
A	73 /M	Pneumonia	19.2	52	Severe LV systolic dysfunction	No	No	Yes
A	70 / F	Gastrointestinal	18.0	47	Moderate LV systolic dysfunction, RV systolic dysfunction	N/A	Yes	No
A	74 / M	CRBSI	16.2	32	Mild LV systolic dysfunction	N/A	Yes	No
A	78 / M	Gastrointestinal	20.3	40	Severe LV systolic dysfunction	Yes	Yes	No
A	77 / M	Undetermined	20.1	50	Mild LV systolic dysfunction	N/A	Yes	No
B	59 / F	Pneumonia	1.5	3	Moderate LV systolic dysfunction	Yes	Yes	No
B	76 / F	Urosepsis	28.8	42	Mild LV systolic dysfunction	Yes	Yes	No
B	84 / M	Gastrointestinal	10.5	14	Severe LV systolic dysfunction	N/A	No	Yes
B	72 / M	Gastrointestinal	10.4	17	Moderate LV systolic dysfunction	N/A	Yes	No
B	74 / M	Pneumonia, Skin/soft tissue	10.4	19	Mild LV systolic dysfunction	N/A	No	Yes
B	83 / M	Pneumonia	11.6	21	Moderate LV systolic dysfunction	Yes	Yes	No
C	70 / M	Pneumonia	42.7	76	Severe LV systolic dysfunction, RV systolic dysfunction	N/A	No	Yes
C	48 / M	Pneumonia	16.0	31	Mild LV systolic dysfunction	Yes	Yes	No
C	58 / M	Skin/soft tissue	162.5	262	Severe LV systolic dysfunction	Yes	Yes	No
C	39 / M	Gastrointestinal	31.8	58	Mild LV systolic dysfunction	N/A	Yes	No
C	86 / F	Pneumonia	21.2	53	Moderate LV systolic dysfunction	N/A	No	Yes
C	58 / M	Gastrointestinal	31.8	76	Moderate LV systolic dysfunction	N/A	No	Yes
D	85 / M	Pneumonia	32.0	70	Mild LV systolic dysfunction	N/A	No	Yes
D	91 / F	Pneumonia	10.8	24	Mild LV systolic dysfunction	N/A	Yes	No
D	86 / M	Pneumonia	53.6	84	Mild LV systolic dysfunction	N/A	No	Yes

Norepi eq: norepinephrine equivalent; VIS: vasoactive-inotropic score; ICU: intensive care unit; LV: left ventricular; N/A: not available; RV: right ventricular; CRBSI: catheter-related bloodstream infection.
